# Amino Acids Supplied through the Autophagy/Endocytosis Pathway Promote Starch Synthesis in *Physcomitrella* Protonemal Cells

**DOI:** 10.3390/plants11162157

**Published:** 2022-08-19

**Authors:** Md. Arif Sakil, Kyosuke Mukae, Ryo Funada, Toshihisa Kotake, Shigeaki Ueno, Most Mohoshena Aktar, Md. Shyduzzaman Roni, Yuko Inoue-Aono, Yuji Moriyasu

**Affiliations:** 1 Graduate School of Science and Engineering, Saitama University, Saitama 338-8570, Japan; 2 Department of Biochemistry and Molecular Biology, Bangladesh Agricultural University, Mymensingh 2202, Bangladesh; 3 Research Institute for Clinical Oncology, Saitama Cancer Center, Saitama 362-0806, Japan; 4 Faculty of Education, Saitama University, Saitama 338-8570, Japan; 5 Department of Agronomy, Hajee Mohammad Danesh Science and Technology University, Dinajpur 5200, Bangladesh; 6 Department of Horticulture, Bangabandhu Sheikh Mujibur Rahman Agricultural University, Gazipur 1706, Bangladesh

**Keywords:** starch synthesis, autophagy, endocytosis, amino acid, *Physcomitrella* (*Physcomitrium*)

## Abstract

The physiological implications of autophagy in plant cells have not been fully elucidated. Therefore, we investigated the consequences of autophagy in the moss *Physcomitrella* by measuring biochemical parameters (fresh and dry weights; starch, amino acid, carbohydrate, and NH_3_ content) in wild-type (WT) and autophagy-deficient *atg5* *Physcomitrella* cells. We found higher starch levels and a higher net starch synthesis rate in WT cells than in *atg5* cells cultured in a glucose-containing culture medium, whereas net starch degradation was similar in the two strains cultured in a glucose-deficient culture medium. Additionally, the treatment of cells with the autophagy inhibitor 3-methyladenine suppressed starch synthesis. Loading bovine serum albumin into *atg5* cells through endocytosis, i.e., supplying proteins to vacuoles in the same way as through autophagy, accelerated starch synthesis, whereas loading glutamine through the plasma membrane had no such effect, suggesting that *Physcomitrella* cells distinguish between different amino acid supply pathways. After net starch synthesis, NH_3_ levels increased in WT cells, although the change in total amino acid content did not differ between WT and *atg5* cells, indicating that autophagy-produced amino acids are oxidized rapidly. We conclude that autophagy promotes starch synthesis in *Physcomitrella* by supplying the energy obtained by oxidizing autophagy-produced amino acids.

## 1. Introduction

In eukaryotic cells, cytosolic components and organelles are degraded in lysosomes and vacuoles continuously and in response to external stresses via a process known as autophagy [1]. Autophagic pathways are classified into macroautophagy, microautophagy, and chaperone-mediated autophagy according to how cytosolic components are transported into lysosomes and vacuoles. Macroautophagy (hereafter referred to as autophagy) has been studied extensively [2] and is known to involve the temporary sequestration of cytoplasmic components in double membrane-enclosed organelles known as autophagosomes, followed by their transport into vacuoles and lysosomes for degradation by resident hydrolases [3]. This process is regulated by approximately 16 core autophagy-related (*ATG*) gene-encoded proteins [4]. Homologs of core *ATGs* are conserved in plants [5,6].

The primary consequences of autophagy are the elimination of cytosolic components and macromolecules and the supply of degradation products, such as amino acids, to cells in which they are used for protein synthesis or energy production [7]. The physiological implications of autophagy, including the degradation of chloroplasts, peroxisomes, and starch granules under nitrogen and carbon starvation conditions [8,9,10,11,12,13], have also been revealed in studies using mainly *Arabidopsis* and rice *atg* mutants. Moreover, autophagy has been shown to prevent early senescence and cell death in *Arabidopsis* [5,14]. Although some of the effects of autophagy are associated directly with the degradation of cellular components, other effects are seemingly not associated with the primary functions of autophagy; thus, the involvement of autophagy in some phenomena has not been fully clarified.

To investigate the physiological effects of autophagy in plant cells, we constructed *ATG* knockout *atg5* mutants in the moss *Physcomitrella patens* because *Physcomitrella* is useful for analyzing the implications of autophagy at the cellular level. Specifically, we examined the physiological consequences of autophagy deficiency using wild-type (WT) and *atg5* mutant *Physcomitrella* colonies. When these colonies were grown on a nutrient-sufficient medium, they had different dry weight (DW) to fresh weight (FW) ratios (DW/FW). We found that starch accumulation was responsible for an increase in DW/FW in WT cells; therefore, we further investigated the involvement of autophagy in starch accumulation. Briefly, we found that *Physcomitrella* cells cultured under nutrient-sufficient conditions perform autophagy that promotes starch synthesis rather than growth.

## 2. Results

### 2.1. DW/FW of atg5 Cells Is Lower than That of WT Cells

**Figure 1:**
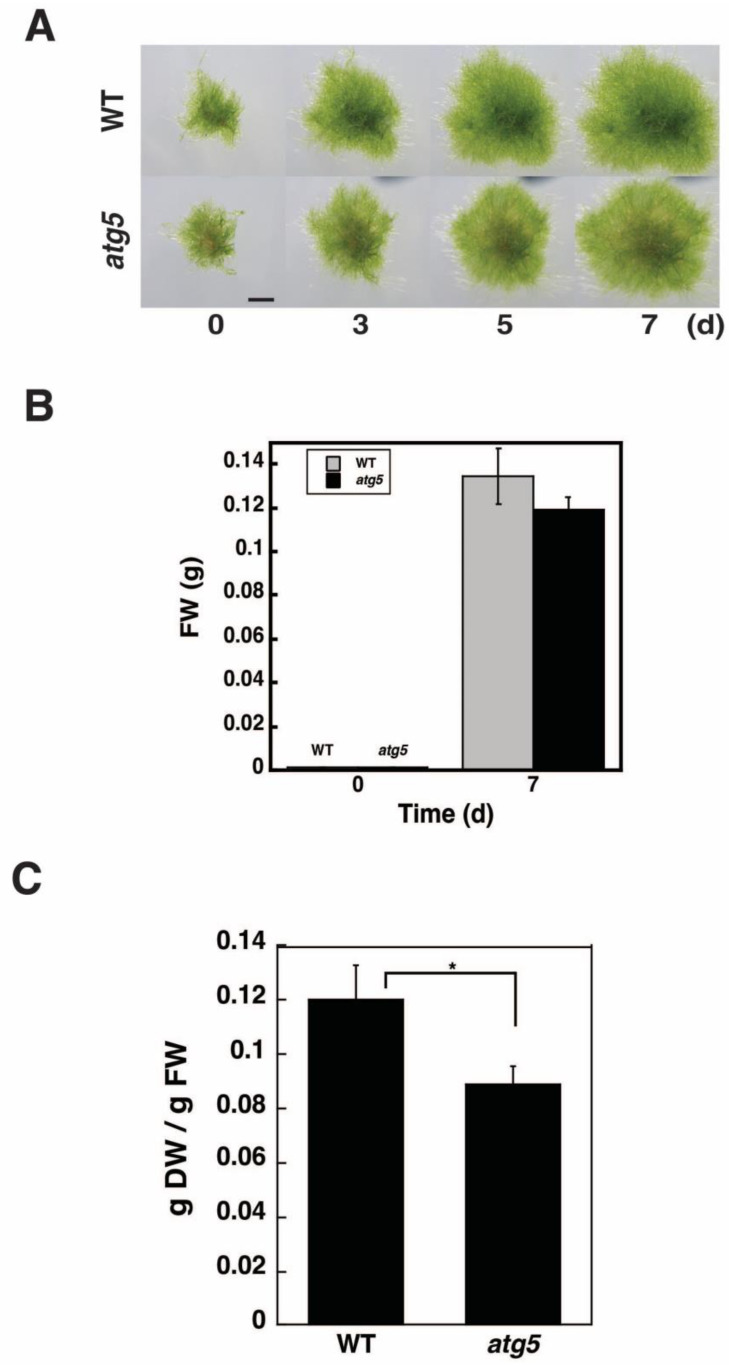
Growth of wild-type (WT) and *atg5* mutant *Physcomitrella* colonies. WT and *atg5* mutant cells of *Physcomitrella* were transferred onto BCDATG agar medium and cultured under light conditions for 7 d. (**A**) The same colonies were photographed immediately (0 d), 3, 5, and 7 d after transfer. Scale bar: 2 mm. (**B**) Fresh weight (FW) of 50 WT and *atg5* colonies was measured immediately (0 d) and 7 d after transfer. (**C**) Dry weight (DW) was also measured, and the DW to FW ratio (DW/FW) was calculated. (**B**,**C**) Data are shown as means ± standard deviation (SD) (*n* = 3, * *p* < 0.05).

WT and autophagy-deficient *atg5* colonies cultured under light conditions for 7 d on nutrient-sufficient BCDATG agar medium were indistinguishable from each other when observed under a stereomicroscope (Figure 1A). The FW of 50 WT and *atg5* colonies was measured immediately after transfer (0 d) and following 7 d of growth (Figure 1B). The 7-day increase in FW did not differ significantly between the WT and *atg5* colonies when they were cultured under nutrient-sufficient conditions (Figure 1B). Thus, on nutrient-sufficient BCDATG agar medium under light conditions, autophagy deficiency does not affect colony growth. In contrast, the DW of the *atg5* mutant colonies was lower than that of the WT colonies. Thus, DW/FW was higher in WT cells than in *atg5* cells (Figure 1C).

### 2.2. Autophagy Deficiency Leads to a Decrease in Starch Accumulation

**Figure 2:**
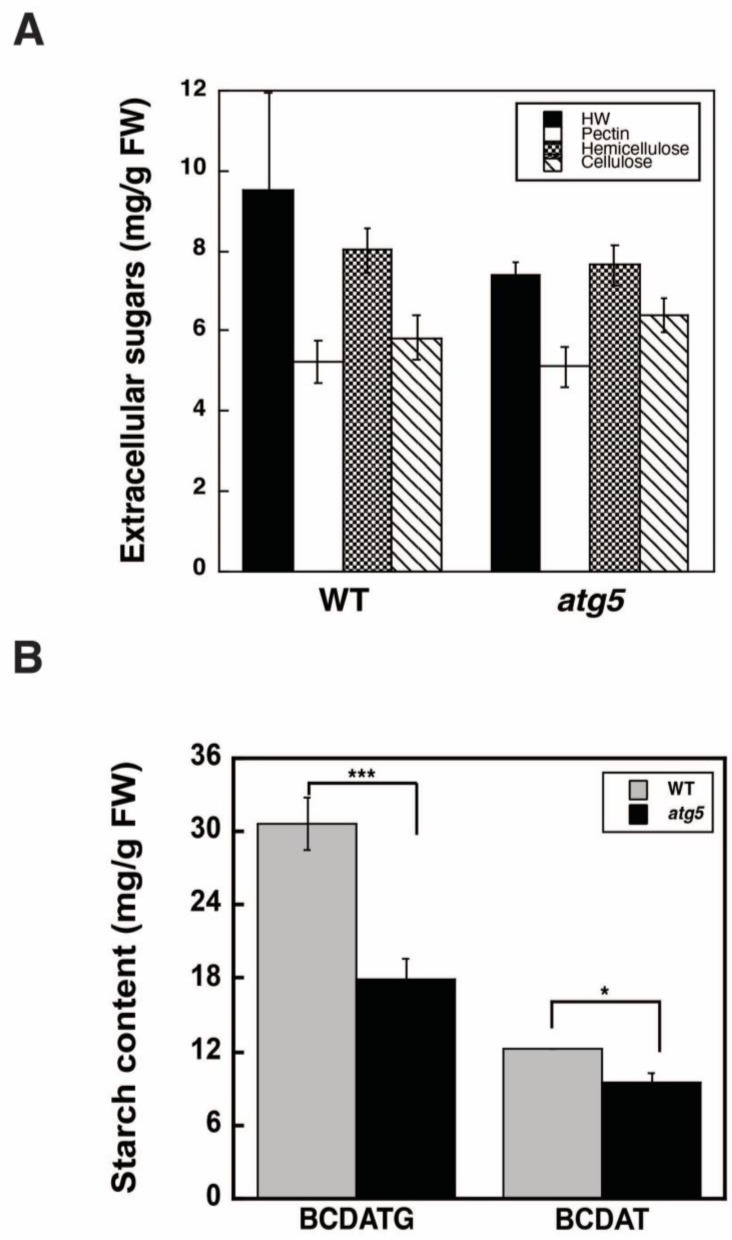
Extracellular sugars and starch in WT and *atg5* mutant *Physcomitrella* cells. (**A**) WT and *atg5* cells were cultured on BCDATG agar medium for 7 d under light conditions. The cell wall components were fractionated into hot water extracted (HW), pectin, hemicellulose, and cellulose fractions, and the carbohydrate content of each fraction was measured. (**B**) Starch content was measured in 7-d-old WT and *atg5* cells cultured on BCDATG or BCDAT agar medium. (**A**,**B**) Data are shown as means ± SD (*n* = 3, *** *p* < 0.005, * *p* < 0.05).

The DW of plant cells mainly comprises carbohydrates; thus, we measured the components of the cell wall. WT and *atg5* colonies were grown on a BCDATG medium for 7 d under light conditions, and the components of the cell wall were fractionated into hot water-extracted, pectin, hemicellulose, and cellulose fractions. Subsequently, the carbohydrate content in each fraction was measured. In the pectin, hemicellulose, and cellulose fractions, there was no significant difference in carbohydrate content between the WT and *atg5* cells; however, in the hot water-extracted fraction, the WT cells had higher carbohydrate content than the *atg5* cells (Figure 2A). The higher carbohydrate content in the hot water-extracted fraction likely reflected contamination from intracellular carbohydrate, i.e., starch; thus, we subsequently measured the starch content in both cell types.

The starch content of WT cells was significantly higher than that of *atg5* cells (Figure 2B). Specifically, the difference in starch content between the two strains reached approximately 12 mg/g FW when they were grown on a BCDATG agar medium containing 0.5% (*w*/*v*) glucose; thus, there was a significant difference in DW between the strains. Indeed, the DW/FW value in the WT strain can be explained partially by the increased accumulation of starch. After being passaged and cultured several times on BCDAT, a glucose-deficient medium, starch content was significantly reduced in both the WT and *atg5* cells; nevertheless, the starch content of the WT strain remained higher than that of the *atg5* strain (Figure 2B).

### 2.3. Starch Synthesis Rate Is Higher in WT Cells than in atg5 Cells

**Figure 3:**
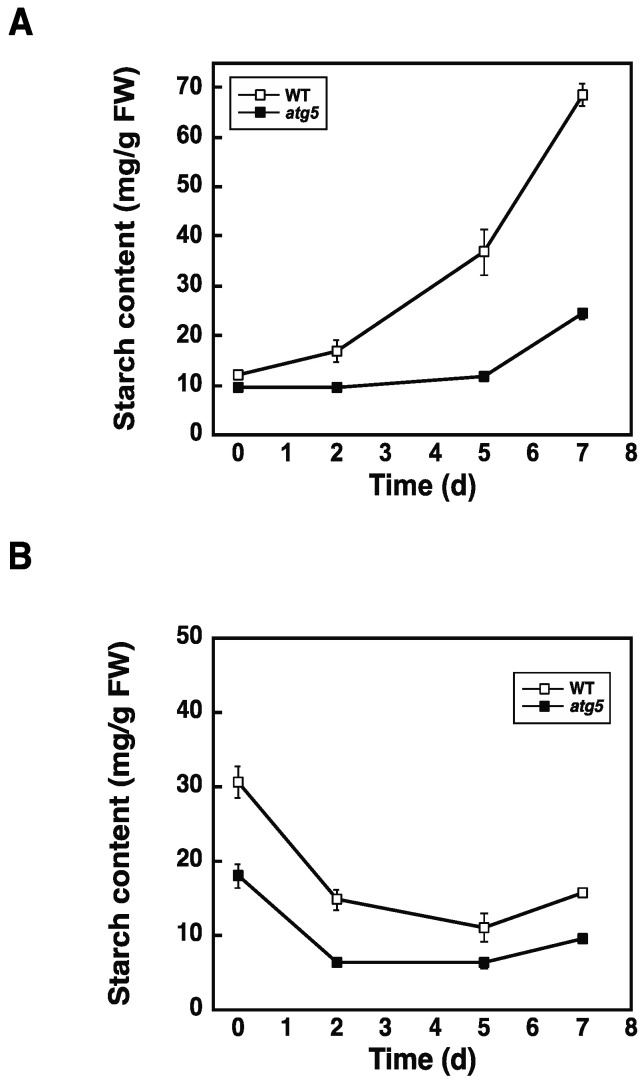
Starch synthesis and degradation in WT and *atg5* mutant *Physcomitrella* colonies. (**A**) Seven-day-old WT and *atg5 Physcomitrella* colonies cultured on BCDAT agar medium were transferred to BCDATG agar medium and cultured for another 7 d. The starch content of colonies was measured for 7 d. (**B**) Seven-day-old WT and *atg5 Physcomitrella* colonies cultured on BCDATG agar medium were transferred to BCDAT agar medium and cultured for another 7 d under light conditions. After transfer, the starch content of the colonies was measured for 7 d. (**A**,**B**) Data are shown as means ± SD (*n* = 3).

To compare the starch synthesis rate between WT and *atg5* cells, colonies cultured on BCDAT agar medium were transferred to BCDATG agar medium and grown for another 7 d (Figure 3A). Over 7 d, starch content increased at a higher rate in WT colonies than in *atg5* colonies, indicating that net starch synthesis was higher in WT cells. Conversely, when WT and *atg5* colonies cultured on BCDATG agar medium were transferred to BCDAT agar medium and cultured for another 7 d, net starch degradation was observed in both strains with no significant difference in the rate of degradation between the two strains (Figure 3B). Therefore, the higher starch content in WT cells than in *atg5* cells is likely due to the higher starch synthesis rate of WT cells.

### 2.4. Inhibition of Autophagy via 3-Methyladenine Decreases the Starch Synthesis Rate

**Figure 4:**
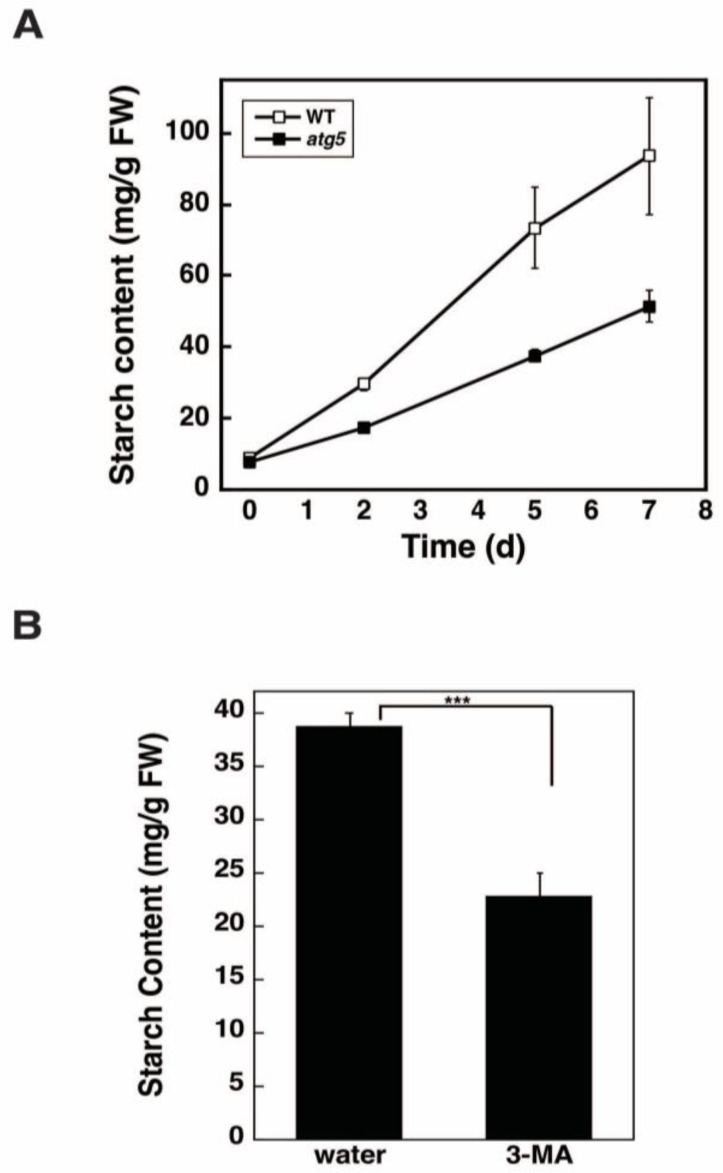
Effects of the autophagy inhibitor 3-methyladenine (3-MA) on starch synthesis. (**A**) WT and *atg5* mutant *Physcomitrella* cells cultured on BCDAT agar medium were transferred to BCDATG liquid medium and cultured for a further 7 d under light conditions, during which their starch content was measured. (**B**) WT *Physcomitrella* colonies grown on a BCDAT agar medium were transferred to a BCDATG liquid medium containing 5 mM of 3-MA or water (as a solvent control) and cultured under light conditions. The starch content of colonies was measured 2 d after transfer. (**A**,**B**) Data are shown as means ± SD (*n* = 3, *** *p* < 0.005).

Having shown that starch synthesis is promoted by autophagy (Figure 3A), we attempted to determine whether this promotion occurs via autophagy that occurs during starch synthesis or before starch synthesis begins. To this end, we used the autophagy inhibitor 3-methyladenine (3-MA) in a liquid culture medium; therefore, we first confirmed that starch synthesis occurs at a higher rate in WT cells than in *atg5* cells following transfer to BCDATG liquid medium (Figure 4A).

Next, we examined the effect of 3-MA on starch synthesis in WT cells. WT colonies cultured on a BCDAT agar medium were transferred to a BCDATG liquid medium containing 5 mM of 3-MA or water (solvent control) and cultured for 2 d. The administration of 3-MA decreased starch synthesis significantly (Figure 4B), suggesting that autophagy that occurs during starch synthesis promotes starch synthesis.

### 2.5. Loading of Protein via the Endocytosis Pathway Promotes Starch Synthesis in atg5 Cells

**Figure 5:**
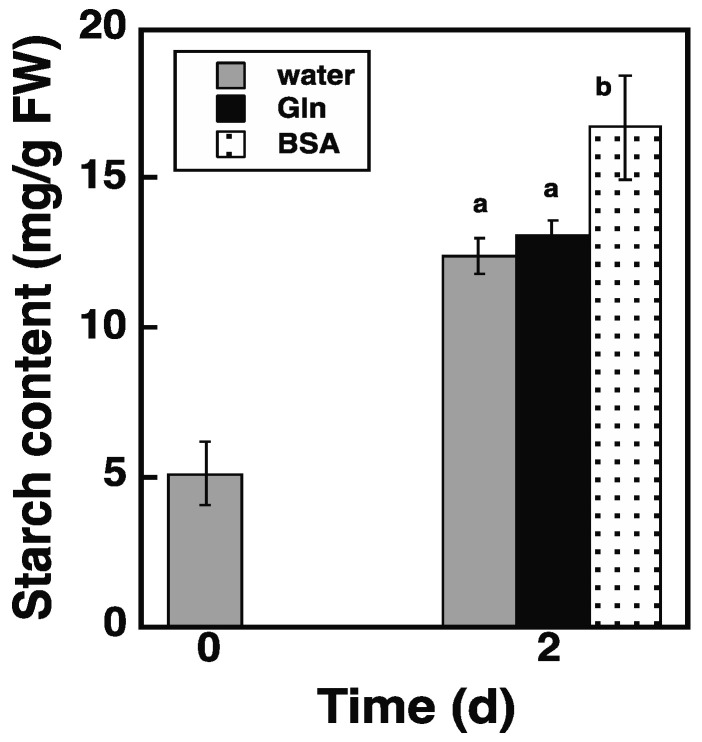
Effects of bovine serum albumin (BSA) and glutamine (Gln) on starch synthesis in *atg5* mutants. *Physcomitrella atg5* colonies cultured on a BCDAT agar medium were transferred to BCDATG liquid medium containing 2% (*w*/*v*) BSA, 1 mM Gln, or water (as a solvent control) and cultured under light conditions. The starch content of colonies was measured immediately (0 d) and 2 d after transfer. Data are shown as means ± SD (*n* = 3). Different letters denote significant differences from each other, *p* < 0.01).

The autophagy and endocytosis pathways merge in the same lytic compartments, e.g., lysosomes and vacuoles, to achieve protein degradation [15,16]. Thus, the endocytosis of bovine serum albumin (BSA) can produce amino acids in vacuoles in a similar manner to the autophagy of intracellular proteins. If autophagy promotes starch synthesis through the production of amino acids, administering BSA should also promote starch synthesis in *atg5* cells. The effects of BSA were assessed using *atg5* colonies cultured on a BCDAT agar medium and transferred to BCDATG liquid medium containing 2% BSA or water (solvent control). After culturing in a BCDATG medium for 2 d, starch content increased in the water control (Figure 5, water) and increased further with the addition of BSA to the medium (Figure 5, BSA). The result shows that BSA promotes starch synthesis in *atg5* cells and suggests that in WT cells, the amino acids produced by autophagy promote starch synthesis.

Glutamine (Gln) is the first amino acid synthesized by nitrogen assimilation in plant cells and provides organic nitrogen to all other amino acids. Therefore, we investigated whether administering Gln could replace the amino acids produced by autophagy. However, the addition of 1 mM of Gln did not further increase the starch content in *atg5* colonies after 2 d of growth compared with that in the water control (Figure 5, Gln). Therefore, *Physcomitrella* cells likely distinguish between the amino acid supply from the autophagy/endocytosis pathway and that from the nitrogen assimilation pathway.

### 2.6. Change in Total Amino Acid Content before and after Starch Synthesis Did Not Differ between WT and atg5 Cells

**Figure 6:**
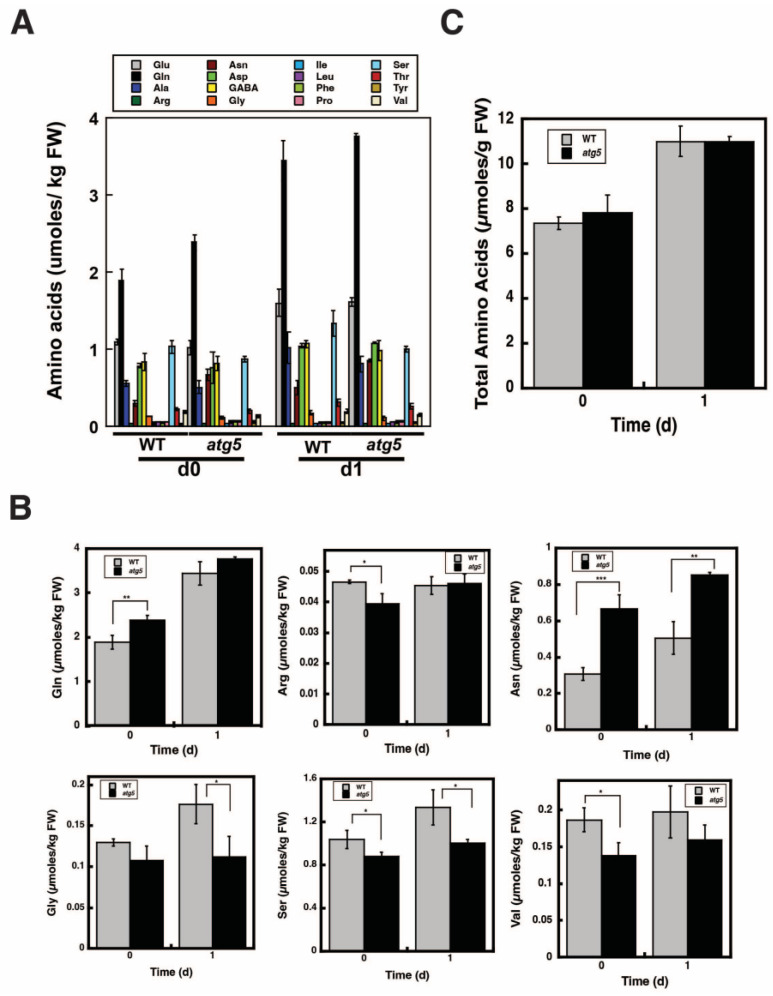
Changes in the amino acid levels in WT and *atg5 Physcomitrella* cells following their transfer from BCDAT to BCDATG medium. WT and *atg5* mutant *Physcomitrella* cells grown on BCDAT agar medium were transferred to BCDATG liquid medium and grown under light conditions. (**A**) Amino acids were extracted from WT and *atg5* cells immediately (0 d) and 1 d after transfer and measured. (**B**) Among the 15 amino acids detected and γ-aminobutyric acid (GABA), the 6 amino acids with significantly different levels in WT and *atg5* cells immediately (0 d) and 1 d after transfer are presented. (**C**) Total amino acid levels i.e., the summation of the 16 amino acid levels immediately (0 d) and 1 d after transfer in WT and *atg5* cells are shown. (**A**–**C**) Data are shown as means ± SD (*n* = 3, * *p* < 0.05, ** *p* < 0.01, *** *p* < 0.005).

The primary functions of autophagy are the degradation of cellular proteins and the production of amino acids; thus, autophagy increases amino acid levels. We compared amino acid levels between WT and *atg5* cells before net starch synthesis, i.e., immediately after 7 d of culture on BCDAT agar medium and 1 d after net starch synthesis on BCDATG agar medium (Figure 6A). Among the 15 amino acids identified and γ-aminobutyric acid (GABA), the levels of 6 amino acids, namely Gln, arginine, asparagine, glycine, serine, and valine, differed significantly between WT and *atg5* cells before and after net starch synthesis (Figure 6B). However, total amino acid levels did not differ significantly between WT and *atg5* cells before and after starch synthesis. Specifically, total amino acid levels increased slightly and by a similar increment in WT and *atg5* cells during 1 d of culture on BCDATG agar medium (Figure 6C). Therefore, we found no evidence to support the occurrence of autophagy at the amino acid level.

### 2.7. Ammonia Content Is Higher in WT Cells than in atg5 Cells

**Figure 7:**
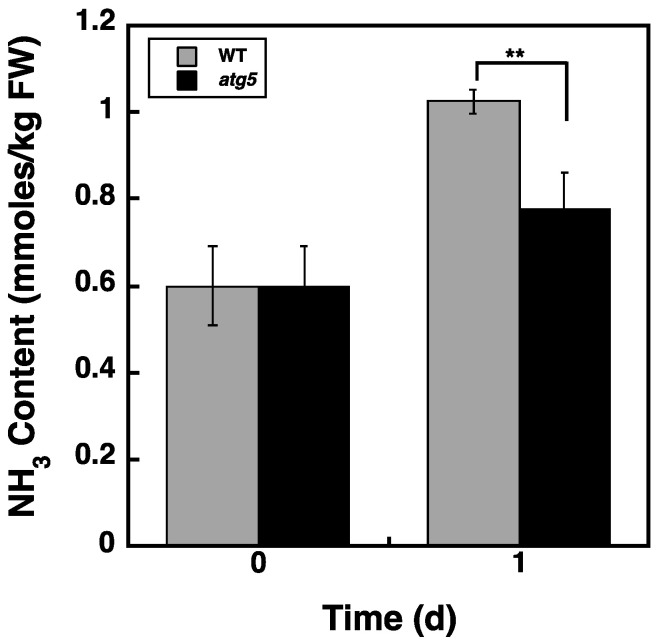
Changes in the ammonia (NH_3_) content of WT and *atg5 Physcomitrella* colonies following their transfer from BCDAT to BCDATG medium. Seven-day-old WT and *atg5 Physcomitrella* colonies grown on BCDAT agar medium were transferred to BCDATG liquid medium and grown under light conditions. NH_3_ content in WT and *atg5* mutants was measured immediately (0 d) and 1 d after transfer. Data are shown as means ± SD (*n* = 3, ** *p* < 0.01).

Considering that we found no significant change in the total amino acid levels due to amino acid production via autophagy (Figure 6C), we hypothesized that the amino acids generated by autophagy are immediately oxidized for energy production. Because the oxidation of amino acids results in an increase in ammonia (NH_3_) in cells, we measured NH_3_ content and found that NH_3_ levels were similar in WT and *atg5* cells cultured on a BCDAT agar medium (Figure 7). After these cells were transferred to BCDATG liquid medium for 1 d, the cellular NH_3_ content increased, and the increase was significantly higher in WT cells than in *atg5* cells (Figure 7). Thus, these results support the notion that amino acids produced through autophagy are oxidized immediately to produce energy during starch synthesis.

### 2.8. Loading of BSA via the Endocytosis Pathway Increases NH_3_ Content in atg5 Cells

**Figure 8:**
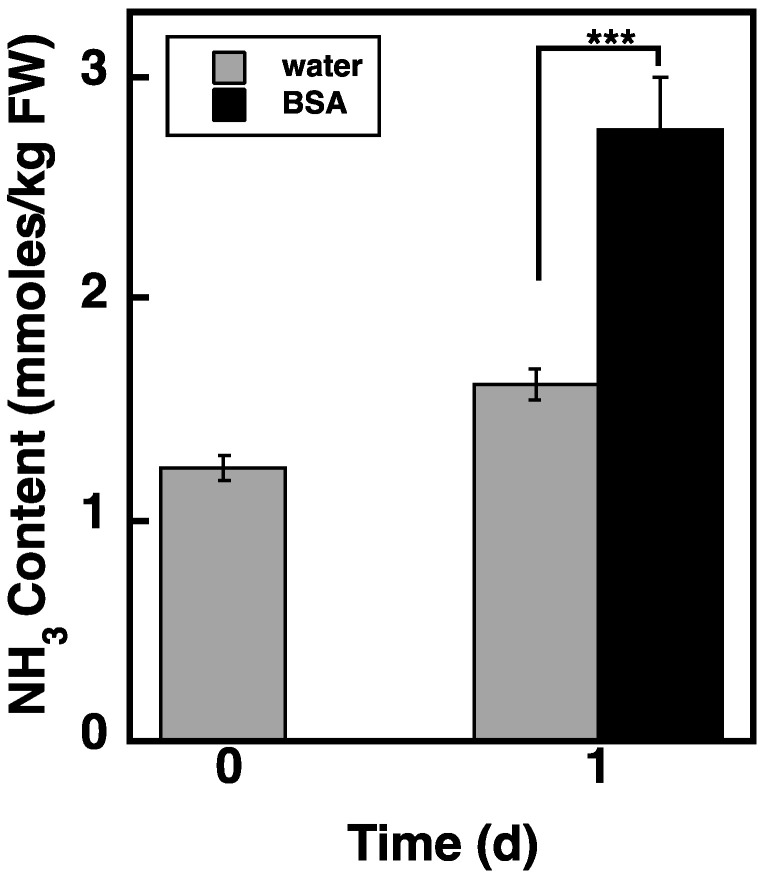
Effects of BSA administration on the NH_3_ content of *atg5* cells. Seven-day-old *atg5 Physcomitrella* colonies grown on a BCDAT agar medium were transferred to BCDATG liquid medium containing 2% (*w*/*v*) BSA or water (as a solvent control) and cultured for 1 d under light conditions. NH_3_ ions were measured immediately (0 d) and 1 d after transfer. Data are shown as means ± SD (*n* = 3, *** *p* < 0.005).

Administration of BSA promoted net starch synthesis in *atg5* cells (Figure 5, BSA). If the amino acids derived from BSA are oxidized to provide energy for starch synthesis, BSA administration should increase NH_3_ content. Therefore, we examined the effects of BSA administration on NH_3_ content in *atg5* cells. After culturing *atg5* cells in a BCDATG medium for 1 d, NH_3_ content increased in the water control (Figure 8, water) and further increased with the addition of BSA to the medium (Figure 8, BSA). This result suggests that a substantial amount of BSA-derived amino acids is oxidized. The energy produced by the oxidation of amino acids is likely promotes starch synthesis in *atg5* cells.

### 2.9. Glucose and Sucrose Levels Do Not Differ between WT and atg5 Cells during Starch Synthesis

**Figure 9:**
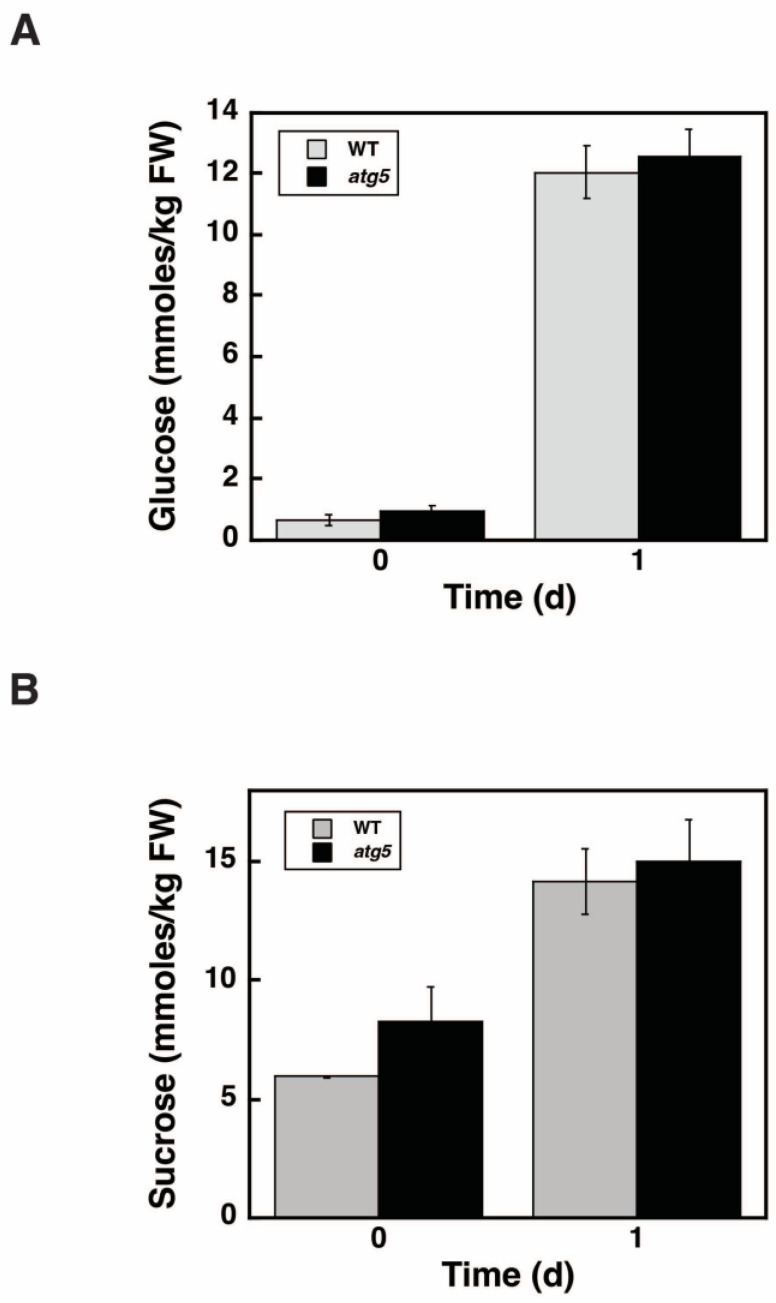
Changes in the glucose and sucrose levels in WT and *atg5 Physcomitrella* colonies following their transfer from BCDAT to BCDATG medium. Seven-day-old WT and *atg5 Physcomitrella* colonies grown on BCDAT agar medium were transferred to BCDATG liquid medium and grown under light conditions. (**A**) Glucose and (**B**) sucrose levels in WT and *atg5* cells were measured immediately (0 d) and 1 d after transfer. (**A**,**B**) Data are shown as means ± SD (*n* = 3).

We also investigated whether autophagy during the starch synthesis affects glucose and sucrose levels. Seven-day-old WT and *atg5* colonies grown on a BCDAT agar medium were transferred to BCDATG liquid medium, and glucose and sucrose levels were measured immediately (0 d) and 1 d after transfer. After 7 d of culture on BCDAT agar medium, glucose (Figure 9A) and sucrose (Figure 9B) levels were <1 mmoles/kg FW and ~7 mmoles/kg FW, respectively, in both WT and *atg5* cells and did not differ significantly between the cell types. The levels of glucose and sucrose both increased 1 d after transfer to BCDATG liquid medium in WT and *atg5* cells, and there was still no significant difference in these levels between the cell types. Therefore, the presence or absence of autophagy does not affect glucose and sucrose levels.

## 3. Discussion

### 3.1. Contribution of Autophagy to Starch Synthesis

In this study, we found that DW/FW differed between WT and *atg5* mutant *Physcomitrella* cells (Figure 1C) and that this difference was due to the starch content of the cells (Figure 2B). We attributed the difference in starch content between WT and *atg5* cells to the difference in their starch synthesis rate and further investigated the promotion of starch synthesis by autophagy. Our findings indicate that autophagy that occurs during starch synthesis promotes such synthesis through the production of amino acids. The treatment of cells with the autophagy inhibitor 3-MA decreased the starch synthesis rate in WT cells (Figure 4B). Conversely, the treatment of cells with BSA increased the rate of starch synthesis in *atg5* cells (Figure 5). These results suggest that amino acids produced by proteolysis through autophagy are involved in starch synthesis. Although we found no evidence to support proteolysis via autophagy, i.e., changes in amino acid levels during starch synthesis, we found that WT cells that performed autophagy had increased NH_3_ content compared with *atg5* cells that did not perform autophagy, suggesting that WT cells immediately degrade the amino acids produced via autophagy. We also showed that the administration of BSA to *atg5* cells increased NH_3_ content, which strongly suggests that amino acids from BSA promote starch synthesis through oxidation and energy supply. Taken together, we conclude that autophagy breaks down cellular proteins; the amino acids produced are oxidized in mitochondria; the energy produced promotes starch synthesis in WT cells. In *Arabidopsis* leaf cells placed in the dark, amino acids produced via autophagy, particularly branched-chain amino acids, are thought to be oxidized in mitochondria to produce energy [17,18].

### 3.2. Amino Acid Supply Route

Although *atg5* cells cannot synthesize starch efficiently, even on a glucose-containing medium, their ability to synthesize additional starch was restored by adding BSA to the culture medium (Figure 5). This suggests that BSA is taken up into cells through the endocytosis pathway and degraded in vacuoles, in which protein degradation and thus amino acid production through the autophagy pathway occur. In contrast to BSA, treatment with Gln did not affect the starch synthesis rate of *atg5* cells (Figure 5), suggesting that *Physcomitrella* cells may distinguish between amino acid supply pathways, perhaps preferring amino acid supply from vacuoles for oxidative degradation in mitochondria and energy production. The physiological importance of amino acid transport from vacuoles to mitochondria for energy production has been discussed in *Arabidopsis* leaf cells placed in the dark [18]. However, the mechanism remains unclear, including whether a particular mechanism exists. In a future study, we will investigate the effects of administering various amino acids on starch synthesis in *atg5* cells.

### 3.3. Involvement of Autophagy in Starch Degradation

It was reported that autophagy contributes to starch degradation in *Arabidopsis* at night [12], although other researchers have observed that starch is degraded in a similar manner between WT and *atg* strains under similar conditions [18]. In *Physcomitrella* cells treated under our experimental conditions, we did not observe the involvement of autophagy in starch degradation (Figure 2B).

## 4. Materials and Methods

### 4.1. Biological Materials

Both WT and autophagy-deficient *atg5* mutant strains [19] of *Physcomitrella* were cultured on BCDATG, containing 0.5% (*w*/*v*) glucose, and BCDAT, which does not contain glucose [20], agar media overlaid with cellophane under continuous light (5–7 w/m^2^ from fluorescent lamps) at 25 °C. Once per week, parts of the colonies consisting of protonemal cells were removed with forceps and placed on a fresh culture medium. Seven-day-old colonies were used in the experiments. The *atg5* strain used in this study corresponds to *atg5*-3 isolated in a previous study [19].

### 4.2. Measurement of FW and DW

Fifty WT or *atg5* colonies from one culture plate were collected with forceps, and their FWs were measured using an analytical balance (Mettler Toledo AG135) after the residual culture medium was removed via blotting on filter paper. The colonies were then homogenized with 600 µL of 0.1 M NaOH and centrifuged at 15,000× *g* for 10 min to extract water-soluble proteins for another purpose. The resulting precipitate was dried at 90 °C for 24 h and weighed using the analytical balance to obtain its DW.

### 4.3. Analysis of Cell Wall Polysaccharides

Fractionation and quantification of cell wall polysaccharides were performed as described previously [21,22]. The colonies were homogenized to a fine powder in liquid nitrogen using a pestle and mortar. The homogenate was washed twice with water and heated in 80% (*v*/*v*) ethanol at 100 °C for 15 min to inactivate endogenous enzymes in the cell wall, after which it was treated with α-amylase (Type VII-A from the porcine pancreas; Sigma, MO, USA) in 50 mM of 3-morpholinopropanesulphonic acid-Na (pH 6.5) at 37 °C for 4 h. Following centrifugation, the cell wall materials were sequentially extracted at 100 °C using water, 50 mM of EDTA (pH 6.8) (pectin fraction), and 17.5% (*w*/*v*) NaOH containing 0.04% NaBH_4_ (hemicellulose fraction). The residual precipitate was washed with water, ethanol, and diethyl ether and collected as the cellulose fraction. The hemicellulose fraction was neutralized using acetic acid, dialyzed against water at 4 °C for 1 d, and lyophilized. The sugar content in each fraction was measured using the phenol–sulfuric acid method, with glucose used as the standard [23].

### 4.4. Measurement of Starch Content

To measure the starch content of a colony, ~0.05 g of the colony was homogenized with 5 mL of 0.1-M acetic-Na (pH 5.0) containing 5 mM of CaCl_2_ using a pestle and mortar. A sample of the homogenate (2 mL) was separated into two microcentrifuge tubes (1 mL per tube). In one tube, starch was hydrolyzed using thermostable α-amylase and amyloglucosidase (Total Starch Assay Kit; Megazyme, K-TSTA-50 A, Wicklow, Ireland) according to the manufacturer’s instructions. In the other tube, the homogenate was maintained without the addition of enzymes. The homogenates were centrifuged at 15,000× *g* for 5 min, and the glucose contents of the two supernatants were measured using a glucose measuring kit (Glucose C2; Wako Pure Chemical Industries, Ltd., Osaka, Japan) (described in more detail below). The difference in glucose content between tubes with and without enzymes was attributed to starch-derived glucose.

### 4.5. Measurement of Amino Acid Levels

To measure amino acid levels in a colony, ~0.1 g of the colony was homogenized with 2 mL of 80% *(v*/*v*) ethanol using a pestle and mortar. The homogenate was centrifuged at 15,000× *g* for 10 min in two microcentrifuge tubes. The precipitate was suspended in 1 mL of 80% (*v*/*v*) ethanol and centrifuged again at 15,000× *g* for 5 min. The supernatants of the first and second centrifugation were pooled and evaporated at 38 °C–40 °C using a rotary evaporator. The dried pellet was dissolved in 1 mL of water and centrifuged at 15,000× *g* for 10 min to remove insoluble materials. The supernatant was then used to analyze amino acids, which were measured using a high-performance liquid chromatography-mass spectrometry (LCMS2010, Shimadzu, Kyoto, Japan) [24].

### 4.6. Measurement of Glucose and Sucrose Levels

Samples prepared for amino acid analysis were also used to measure glucose and sucrose levels. The samples were diluted with water (100 μL) appropriately and added to two separate test tubes (13 × 100 mm). One test tube was filled with 20 μL of buffer 30 mM of acetic-Na [pH 5.5] containing invertase (Invertase from baker’s yeast; Sigma, Kawasaki, Japan, 14,504) and incubated at 37 °C for 15 min to allow sucrose hydrolysis. The other test tube was filled with 20 μL of buffer only. Thereafter, glucose was measured using a glucose measuring kit (Glucose C2; Wako Pure Chemical Industries, Ltd., Japan). A_505_ was measured using a spectrophotometer (Smartspec plus, Bio-Rad Laboratories, Inc., Hercules, CA, USA). The difference in glucose levels between tubes with and without invertase was attributed to sucrose-derived glucose.

### 4.7. Measurement of NH_3_ Content

To measure the NH_3_ content in a colony, ~0.05 g of the colony was homogenized with 1 mL of 25-mM H_2_SO_4_ using a pestle and mortar, after which it was centrifuged at 15,000× *g* for 5 min. The NH_3_ content in the supernatant was measured using the NH_3_ Assay Kit (Fujifilm, Wako Pure Chemical Industries Ltd., Japan), after which A_630_ was measured. Commercially available NH_4_NO_3_ (Nacalai Tesque Inc., Kyoto, Japan) was used as the standard.

### 4.8. Statistical Analysis

Statistical analysis was performed using KaleidaGraph (Synergy, Stroudsburg, PA, USA). Two groups were compared using Student’s *t*-test, whereas multiple group comparisons were performed using ANOVA with Tukey’s test. Differences were considered statistically significant at * *p* < 0.05, ** *p* < 0.01 or *** *p* < 0.005.

## 5. Conclusions

Under nutrient-sufficient conditions, *Physcomitrella* cells perform autophagy to promote starch synthesis rather than growth. Furthermore, autophagy produces amino acids that are rapidly oxidized to generate the energy required for starch synthesis.

## Data Availability

All datasets generated for this study are included in the article and further inquiries can be directed to the corresponding author.
